# ^19^F Solid‐State NMR and Vibrational Raman Characterization of Corticosteroid Drug‐Lipid Membrane Interactions

**DOI:** 10.1002/cplu.202100385

**Published:** 2021-11-02

**Authors:** Bethany Mapley, David Townsend, John Griffin, Lorna Ashton, David A. Middleton

**Affiliations:** ^1^ Department of Chemistry Lancaster University Lancaster LA1 4YB United Kingdom; ^2^ Materials Science Institute Lancaster University Lancaster LA1 4YB United Kingdom

**Keywords:** drug delivery, lipid bilayers, phospholipid vesicles, Raman spectroscopy, solid-state NMR

## Abstract

Drug interactions with phospholipid bilayers underpin their behaviour in cell membranes and in liposomal delivery formulations. Liposomal drug delivery in ocular medicine can overcome the physical barriers of the eye and better enable the active molecule to reach its target. Here, Raman and ^19^F solid‐state NMR spectroscopy are used to characterise the interactions of two ocular corticosteroid drugs, difluprednate (DFP) and fluorometholone (FML), with multilamellar vesicles of phosphatidylcholine (PC). ^31^P NMR confirms that the lipid bilayer tolerates a high drug concentration (a drug: lipid molar ratio of 1 : 10). The ^19^F NMR spectra of the drugs in lipid bilayers reveal that FML and DFP have different average orientations within the lipid bilayer. Raman spectra of dried lipid films reveal that PC separates from DFP but not from FML, the less lipophilic of the two drugs. This combined approach will assist the design of, and inform the development of, improved liposomal preparations.

## Graphical abstract

**Vibrational Raman and** ^**19**^ **F NMR spectroscopy** are used to characterize phospholipid vesicles carrying the ocular corticosteroid drugs difluprednate (DFP) and fluoromethalone (FML). NMR chemical shift and ^19^F‐^1^H dipolar couplings are used to determine the average orientation of DFP and Raman spectra of dried lipid films reveal that the lipid partially separates from DFP but not from FML, the less lipophilic of the two drugs. The combined power of the two techniques in liposomal formulation is demonstrated.

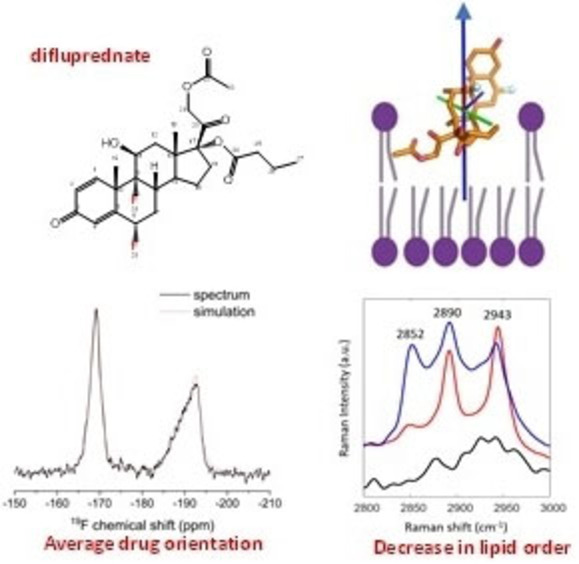

## Introduction

Understanding the structural and dynamic properties of lipophilic and amphiphilic pharmaceuticals within phospholipid bilayers is fundamentally important for assessing their bioavailability and pharmacokinetics and for optimising liposomal drug delivery systems.^[1]^ The partitioning of drugs into cellular membranes, diffusion within the lipid bilayer and subsequent egress into the cellular environment all influence their ability to reach the pharmacological targets.^[2]^ Drug encapsulation by liposomes can be advantageous for improving cellular uptake, enhancing biodistribution and increasing drug stability, and has impacted many areas of biomedicine.^[3]^ One therapeutic area in which liposomal drug delivery has attracted interest is in ocular medicine, as a means of overcoming several physical barriers to drugs reaching their targets on the anterior and posterior of the eye.^[4]^ In order for drugs to be absorbed into the eye, effective corneal penetration and prolonged contact are required, both of which liposomal drug delivery can enhance due to the bioavailability and low toxicity of liposomes.^[5]^ Intravitreal injection of drug‐loaded liposomes has been shown to significantly increase the available concentrations and therapeutic half‐life of drugs in the eye.^[6]^ For example, corticosteroids drugs for treatment of edema, inflammation and angiogenic eye diseases have been delivered to the eye with nanostructured lipid carriers.^[7]^

**Figure 1:**
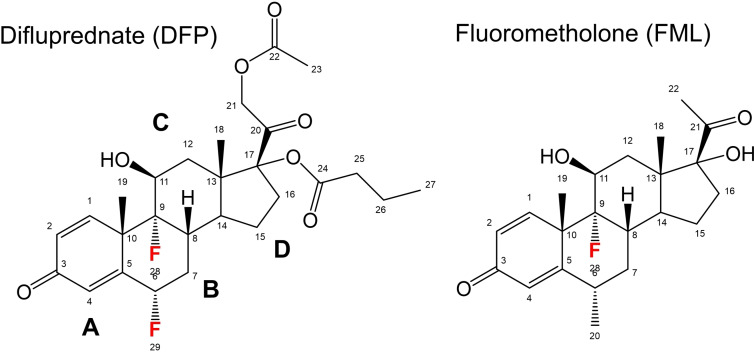
Chemical structures of two corticosteroid drugs. The steroid rings are labelled **A**‐**D**.

In this work, ^19^F solid‐state NMR and vibrational Raman spectroscopy are used to examine the interactions of two corticosteroid drugs (Figure 1) with model palmitoyloleoylphosphatidylcholine (POPC) multilamellar vesicles (MLVs). Difluprednate (DFP) is a difluorinated drug used for the treatment of post‐operative ocular inflammation and pain and was approved by the US Food and Drug Administration in 2008. Fluoromethalone (FML) is another ocular anti‐inflammatory drug, which has the same steroidal ring structure as DFP but with a single fluorine substituent and a shorter aliphatic tail. ^19^F NMR spectra on hydrated vesicles report the average orientation of the drugs within lipid bilayers and their effects on the structure and stability of the lipid bilayer. Raman analysis is carried out on films of dried lipid vesicles containing the drugs, to establish how the drying process (used to preserve liposome stability) affects the drug distribution. This information is of use when developing new lipid formulations of the drug.

## Results and Discussion

### Solid‐state NMR analysis of DFP in hydrated vesicles

**Figure 2:**
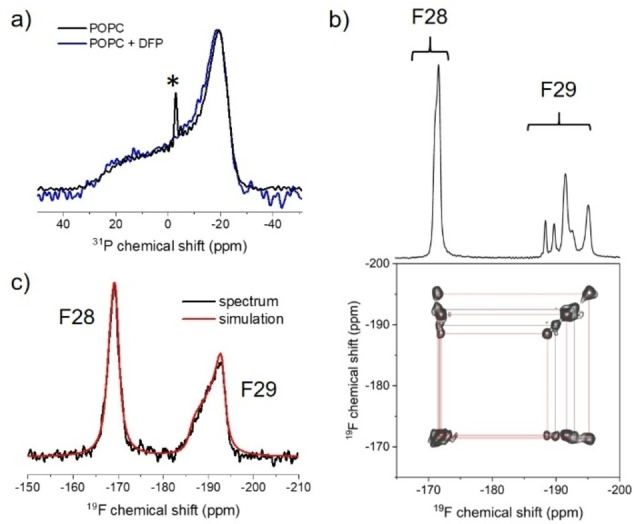
NMR analysis of DFP in hydrated POPC bilayers. (a) Static ^31^P NMR spectra of POPC membranes alone (black) and in the presence of DFP at a lipid: drug molar ratio of 10 : 1 (blue). The asterisk denotes a small narrow component in the POPC‐only spectrum (<3 % of the overall signal) attributed to small, rapidly tumbling lipid assemblies that often form spontaneously during sample preparation. (b) 2D ^19^F‐^19^F dipolar correlation spectrum and horizontal projection of solid DFP at a MAS frequency of 12 kHz. (c) Static, proton‐decoupled ^19^F NMR spectrum of 10 : 1 POPC:DFP membranes overlaid with the best fitting simulated spectra, from which were obtained values of the motionally‐averaged anisotropy, Δδ_av_, of −1.8 ppm for F28 and +4.3 ppm for F29.

Static ^31^P solid state NMR was used to establish whether the DFP is accommodated within the lipid bilayers without disrupting the lamellar organisation of the vesicles. Spectra of hydrated POPC vesicles alone and with a 10‐fold molar excess of DFP (Figure 2a) exhibit line shapes that are typical for a lipid bilayer.^[8]^ The virtually identical widths (∼45 ppm) and lineshapes of the two spectra indicate that DFP does not perturb the lipid headgroups significantly and that the MLVs tolerate a high concentration of the drug without disruption of the overall lamellar structure. The two fluorine substituents of the steroid ring enable ^19^F NMR characterisation of the behaviour of the drug within the lipid bilayers. The 2D magic‐angle spinning (MAS) ^19^F NMR spectrum of pure solid DFP exhibits several overlapping peaks at around −172 ppm assigned to F28 and fully‐ or partially‐ resolved peaks around −192 ppm assigned to F29 (Figure 2b). The F28–F29 cross‐peaks resolve at least 5 pairs of chemical shifts (Table S1), consistent with multiple crystalline forms of the drug having different conformations.^[9]^ The averages of the measured static powder chemical shift anisotropy (CSA) values, Δ*δ_st_*, for F28 and F29 are 19.4 ppm and −30.2 ppm (Table S2). The ^19^F NMR spectrum of DFP in POPC membranes (obtained without sample spinning) exhibits single peaks for F28 and F29 with substantially reduced chemical shift anisotropies compared to the solid state (Figure 2c and Table S3). The peak narrowing signifies rapid anisotropic averaging of the chemical shift tensors (Figure S1), by motions including internal conformational fluctuations and rotational diffusion of the drug within the lipid bilayer. Peak fitting yielded the ^19^F chemical shift parameters for F28 and F29 (Figure 2c and Table S3), including the dynamically‐averaged CSA values, δΔ_av_. An asymmetry parameter η of zero for both nuclei indicates a symmetrical CSA tensor as a result of nanosecond rotational diffusion about an axis that is, on average, parallel with the bilayer normal. Further, the absence of sharp isotropic components to the line shapes confirms that that DFP partitions fully into the anisotropic environment of the lipid bilayer.

### The average orientation of DFP in POPC bilayers

**Figure 3:**
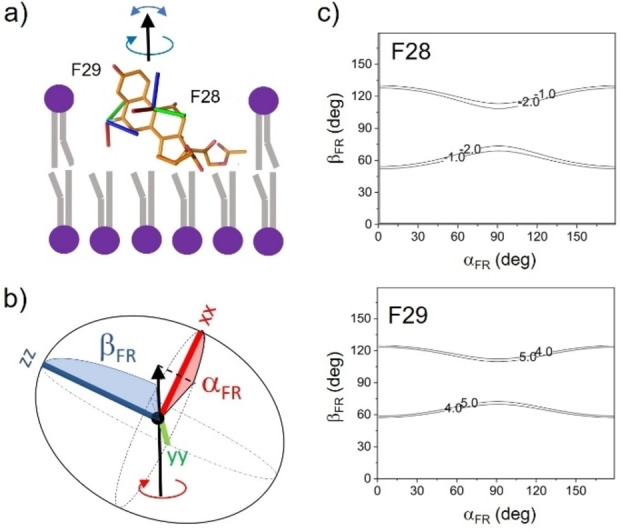
Analysis of the individual ^19^F chemical shift data for DFP in hydrated POPC membranes. (a) The drug undergoes anisotropic rotation about a principal axis (black arrow) that is on average parallel with the bilayer normal, and additional motional fluctuations or “wobble” of the rotation axis away from the bilayer normal represented by order parameter S_mol_. The predicted orientations of the F28 and F29 chemical shift principal axes are represented by red, green and blue lines. (b) Angles α_FR_ and β_FR_ define the orientation of the rotational axis relative to the principal axes xx, yy and zz of the asymmetric ^19^F chemical shift tensors for F28 and F29. (c) Contour plots in which the solid lines indicate the experimentally consistent ranges of Δδ_av_ values (−2.0 ppm≥Δδ_av_≥−1.0 ppm for F28 and 5.0 ppm≥Δδ_av_≥4.0 ppm for F29) calculated from combinations of α_FR_ and β_FR_ angles. An order parameter, *S*_mol_, of 0.8 is assumed.

Further analysis of the ^19^F chemical shift parameters for DFP in lipid bilayers was carried out to determine the dynamically averaged orientation of the drug. The ^19^F NMR line shapes of fluorinated molecules in lipid bilayers are sensitive to their average orientation about the main axis of rotational diffusion.^[10]^ The measured anisotropy, Δδ_av_, is a function of azimuthal angle α_FR_ and polar angle β_FR_, which define the orientation of the molecular rotational axis in a given ^19^F CSA principal axis system (Figure 3, a and b). The relationship is given by^[11]^

(1) $$\Delta \delta {}_{\mathrm{av}}=0.5S{}_{\mathrm{mol}}\;\Delta \delta {}_{\mathrm{st}}\;\left(3\cos{}^{2}\;\beta -1-\eta \sin{}^{2}\beta {}_{\mathrm{FR}}\cos2\alpha {}_{\mathrm{FR}}\right)$$

*S*_mol_ is an order parameter representing the amplitude of excursions of the rotational axis from the bilayer normal by angle *ϕ*, where *S*_mol_=cos *ϕ*. Any pair of [α_FR_, β_FR_] angles can be translated into a particular drug orientation in the bilayer if it is known how the ^19^F chemical shift tensor principal axes, *xx*, *yy* and *zz*, are directed relative to the molecular geometry. The principal axis orientations were here determined from density functional theory calculations on the DFP conformation from the crystal structure after optimisation. It was assumed that the crystal structure represents the average conformation of the drug within the lipid bilayer. The static and averaged ^19^F CSA parameters, measured by spinning side‐band fitting to the solid‐state NMR spectrum at 5 kHz MAS (Table S3; Figure S2), were substituted into Eq [1] and α_FR_ and β_FR_ were each varied from 0–180° to find the angles that give Δδ_av_ values close to those measured for F28 (−1.8 ppm) and F29 (4.3 ppm). Whilst a restricted range of β_FR_ values are calculated for F28 and F29, all possible values of α_FR_ are permitted (Figure 3c). Analysis of Δδ_av_ for each ^19^F nucleus independently of the other cannot therefore determine the drug orientation.

**Figure 4:**
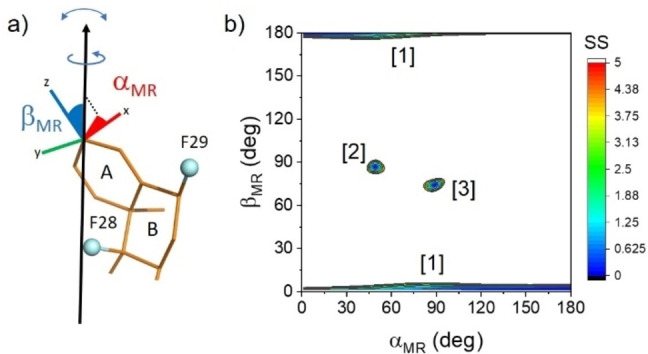
Restricted molecular orientations of DFP in hydrated POPC bilayers obtained by combined analysis of the calculated Δδ_av_ values for F28 and F29. (a) An arbitrary molecular coordinate system is defined relative to the geometry of DFP (only rings A and B are shown for clarity). The orientation of the rotational axis in the molecular frame is defined by angles α_MR_ and β_MR_. (b) All possible orientations of the rotational axis in the molecular frame are sampled by varying α_MR_ and β_MR_ from 0–180°, and Δδ_av_ values for F28 and F29 are calculated for each orientation. Contoured regions show sum‐of‐square (SS) values that represent acceptable agreement between the calculated and measured Δδ_av_ values (SS<5.0 ppm^2^). An order parameter, S_mol_, of 0.8 is assumed.

**Figure 5:**
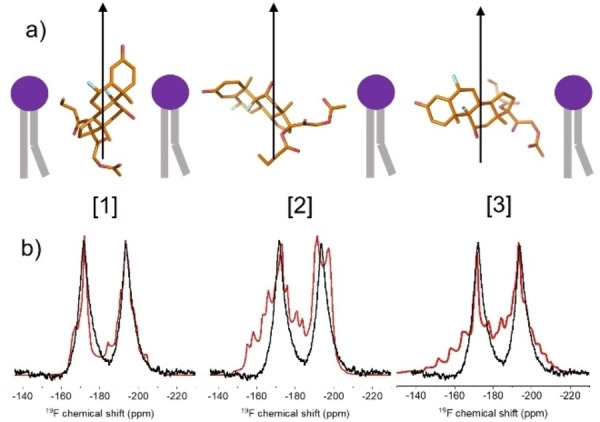
Determination of the average molecular orientation of DFP in hydrated POPC bilayers. (a) Representative orientations from each group, [1], [2] and [3], relative to the rotational axis (blue arrow), which is parallel with the bilayer normal. The axis of rotation is shown in the ^19^F CSA principal axis system for F29, with the origin at the molecular centre of mass. (b) Proton‐coupled static ^19^F spectrum of DFP in POPC bilayers (black) overlaid with simulated proton‐coupled line shapes (red) for the average orientations shown above each spectrum.

An alternative approach was used in which the F28 and F29 CSA data were analysed simultaneously to exploit the different orientations of the F28 and F29 chemical shift tensor axes with respect to the molecular geometry. An arbitrary molecular coordinate system was defined in which the z axis lies along the C3=O bond and the x axis is normal to the plane of ring A (Figure 4a). The orientation of the principal rotation axis in this new reference frame is defined by angles α_MR_ and β_MR_. These angles were varied from 0–180° and, for each orientation, Δδ_av_ was calculated for F28 and F29. The contour plot in Figure 4b shows the angle combinations giving calculated Δδ_av_ values for both F28 and F29 within an acceptable range of the measured values. It is seen that there are just 3 distinct groups of drug orientations that are consistent with the measured Δδ_av_ values for F28 and F29. Group [1] is defined by β_MR_ values of 0°±5°, but α_MR_ takes all possible values. However, because the rotation axis is close to the z axis of the reference frame, the uncertainty in α_MR_ has little impact on the drug orientation, which is upright in the lipid bilayer (Figure 5a). In orientations [2] and [3], the fused ring system of the drug is approximately perpendicular to the bilayer normal (Figure 5a). With the exception of α_MR_ for group [1], the range of angles describing each orientational group covers about 10°. The spread of angles depends to some extent on the choice of S_mol_ in the calculation. A value of 0.8 was used in this case, but lower or higher values are seen to decrease or increase the certainty in the orientation (Table S4).

It is intuitive to assume that group [1] represents the most favourable average orientation of DFP because the volume occupied by the drug in an upright position is less likely to disrupt the lipid bilayer than if the drug were to penetrate the bilayer perpendicularly to the bilayer normal. However, groups [2] and [3] cannot be ruled out from the chemical shift data alone. To attempt to identify the correct orientation from the three groups, orientationally‐dependent ^1^H‐^19^F dipolar couplings were analysed from the lineshape of a proton‐coupled ^19^F NMR spectrum (Figure 5b). The line shapes of the two peaks for F28 and F29 are influenced by intramolecular ^1^H‐^19^F dipolar couplings, *d*_av_, which are averaged by rotational diffusion according to^[19]^

(2) $$d{}_{\mathrm{av}}=0.5\;d{}_{\mathrm{st}}\left(3\cos{}^{2}\theta \;-1\right)$$

The static dipolar coupling, *d*_st_, is dependent on the ^1^H‐^19^F distance and *θ* is the angle between the dipolar vector and the rotational axis. Simulated line shapes were calculated for F28 and F29 using the same CSA parameters in Table S1 but including dipolar couplings to all protons within 5 Å of each ^19^F nucleus (i. e., corresponding to d_st_ values of 904 Hz or higher). The simulation for orientation [1] is clearly in much closer agreement with the spectrum than are the simulations for the other orientations (Figure 5b) and hence the spectrum is consistent with the favoured upright orientation of the molecule.

#### Solid‐state NMR analysis of FML in hydrated vesicles

**Figure 6:**
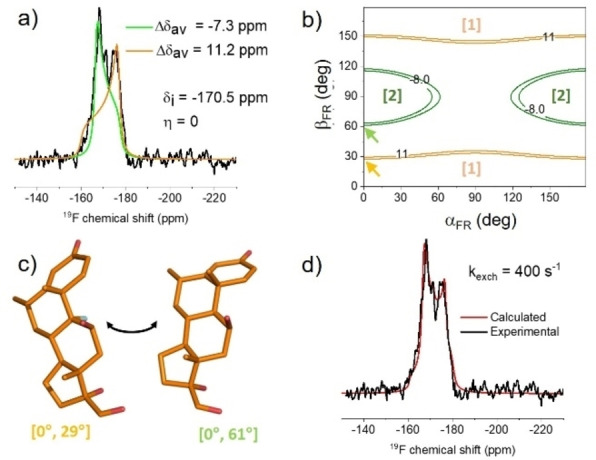
Proton‐decoupled ^19^F NMR analysis of FML in hydrated POPC bilayers. (a) Experimental spectrum (black) overlaid with simulated axially symmetric line shape components (orange and green) correponding to a single isotropic chemical shift, δ_i_, but different values of the averaged chemical shift anisotropy, Δδ_av_. (b) Plot of α_FR_ and β_FR_ angles consistent with the Δδ_av_ values of the two components. The orange and green contours represent Δδ_av_ ranges of −8.0 to −7.0 ppm and 11.0 to 12.0 ppm, respectively. (c) One example of the exchange of FML between orientations, defined by [α_FR_, β_FR_] angles that are consistent with the NMR data. (d) Refined fit to the experimental spectrum after including exchange between the two orientations at a rate constant k_exch_=400 s^−1^ in the lineshape simulation.

The ^19^F spectrum of FML in POPC bilayers (10 : 1 drug: lipid molar ratio) exhibits an unusual multi‐component lineshape centred at around 170 ppm (Figure 6a). The ^31^P NMR spectrum (not shown) is virtually identical to that of the DFP:POPC preparation (Figure 2a), indicating that that the bilayer structure is retained in the presence of the drug. The spectrum can be approximated by two lineshape components (Figure 6a, green and orange lines) calculated from the same isotropic chemical shift and asymmetry parameter (η=0, indicating anisotropic rotation about a principal axis), but different values of Δδ_av_. These two dynamically averaged values are consistent with two average orientations of the drug as defined by angles α_FR_ and β_FR_. The uncertainties in the angles defining the two orientations are considerable (Figure 6b) but cannot be reduced in the same way as for DFP because FML carries only a single fluorine. Taking one possible [α_FR_,β_FR_] combination for each orientation, [0°,29°] and [0°,61°] (Figure 6c), it was found that a closer fit to the experimental lineshape could obtained by assuming exchange between the two orientations (Figure S3). A flattening of the outer wings is seen with increasing exchange rate and a narrow line appears at the isotropic chemical shift. The best fit corresponded to an exchange rate constant, *k*_exch_, of 400 s^−1^ (Figure 6d).

#### Raman analysis of DFP and FML in dried lipid films

**Figure 7:**
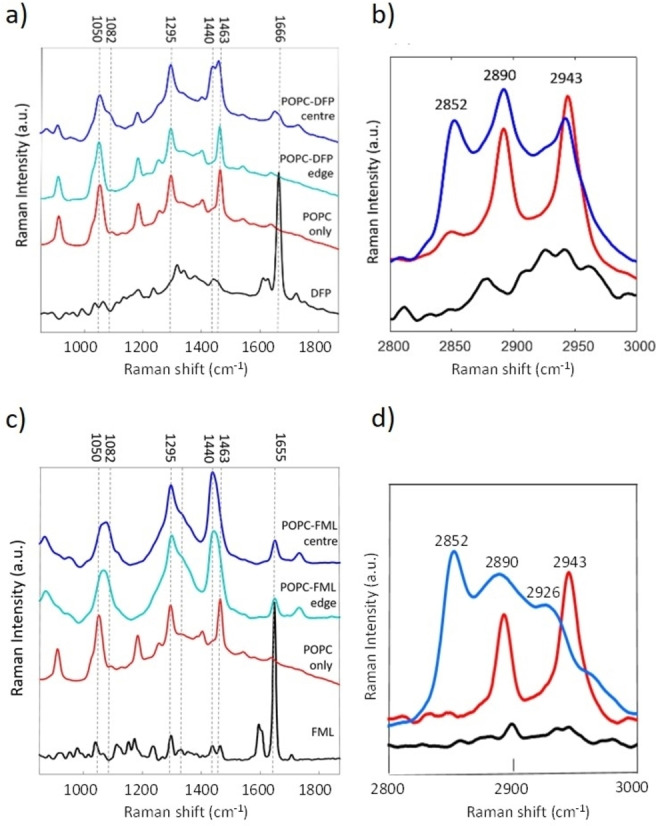
(a) Average Raman spectra (n=8) of DFP (black) and dried films of POPC (red) and POPC‐DFP taken from the thin film edge (cyan) and the central (blue) regions in the wavenumber range 800–1800 cm^−1^. (b) Average Raman spectra (n=8) of DFP (black), POPC (red) and POPC‐DFP complex (blue) in the wavenumber rang 2800–3000 cm^−1^. (c) Average Raman spectra (n=8) of FML (black), POPC (red) and POPC‐FML taken from the thin film edge (cyan) and the central (blue) regions in the wavenumber range 800–1800 cm^−1^. (d) Average Raman spectra (n=8) of FML (black), POPC (red) and POPC‐FML complex (blue) in the wavenumber rang 2800–3000 cm^−1^.

Figure 7a compares the Raman spectra of POPC multilamellar vesicle (MLV) samples prepared as dried films with and without DFP (10 : 1 lipid : drug molar ratio) and the pure spectrum of DFP in the fingerprint wavenumber range 850–1800 cm^−1^. The spectrum for DFP is similar to previously reported spectra^[13]^ and it was hoped that signature peaks in the range 600–800 cm^−1^ corresponding to C−F vibrations could be used to identify the drug within the MLV sample, but these were too weak to be of value. Peaks in the region 1600–1680 cm^−1^ have been assigned to ring A C=C and C=O vibrations in the Raman spectrum of the related compound cortisone and an intense peak can be observed at 1666 cm^−1^ for pure DFP.^[14]^ As expected during thin film formation, for both the POPC and DFP MLV samples a coffee ring effect was observed.^[15]^ In the case of the POPC‐only sample, no difference in spectra, whether collected from the central region or the edge of the film, was observed (see Supplementary Information, Figure S4), However, spectra of the POPC‐DFP sample acquired from the central areas of the film differed from those acquired at the edge (example spectra shown in Figure 7a). The average spectrum acquired from the outer edges of the film is identical to the spectrum of POPC alone, regardless of position, potentially suggesting MLVs without DFP encapsulated have diffused to the outer edges of the thin film.

The dominating DFP peak observed at 1666 cm^−1^ is not present in POPC‐DFP edge spectrum, although a slight shoulder can be observed in the POPC‐DFP centre spectrum it is difficult to conclusively determine the presence of DFP from this peak alone. An additional peak at 1440 cm^−1^ assigned to alkyl CH_2_ bend^[15]^ can be observed in the DFP only spectrum and may be a stronger indicator of the presence of liposome and drug in the central region of the film. A further peak at 1082 cm^−1^ can only be observed in the spectrum of MLVs with DFP encapsulated possibly indicating a change in the liposome conformation as they are not present in the pure DFP spectrum. The peak at 1082 cm^−1^ is assigned to alkyl C−C *gauche* stretch whilst the peak at 1050 cm^−1^ observed in the POPC only spectrum is assigned to the C−C *trans* stretch and have previously been used to assess the phase state of constituent lipids.^[16]^ In Figure 7a, the observed intensity decrease in the peak a 1050 cm^−1^ combined with the appearance of the peak at 1082 cm^−1^ potentially indicates a loss of liposome order with the addition of DFP, although this could be a combined effect of the drug and sample drying, which may destabilise the vesicles. Figure 7b compares the Raman spectra of DFP, POPC and the POPC with encapsulated DFP MLVs in the higher wavenumber range 2800–3000 cm^−1^. No intense Raman peaks can be observed for DFP whilst two intense Raman peaks can be observed for the POPC only sample at 2890 cm^−1^ (CH_2_ asymmetric stretch) and 2943 cm^−1^ (CH_3_ methyl symmetric stretch). Interestingly, the peak observed at 2852 cm^−1^ (CH_2_ symmetric stretch) in the POPC‐DFP sample is far more intense compared to the spectrum of POPC only acquired from the dried films, with a reduced intensity for the peaks at 2943 cm^−1^. It has previously been reported that the ratio of intensities between the symmetrical and asymmetrical methylene stretch peaks is sensitive to intermolecular packing as well as inter‐chain interactions.^[17]^ Sassi et. al^[18]^ also reported a similar change in peak intensity during heating of phosphatidylcholine assigning this to an increase of *gauche* conformer fraction or a loss of lateral packing of the acyl chains. Consequently, the Raman spectra of the POPC‐DFP complex compared to POPC alone indicates a loss of lateral spacing of the acyl chains in the presence of DFP.

Figure 7 (panels c and d) compares the Raman spectra of POPC MLV samples with and without FML. The FML‐only spectrum reveals an intense peak at 1655 cm^−1^ which can be observed as a weaker peak in the spectra acquired from the centre and edge of the film. Unlike the POPC‐DFP spectrum, no difference in the spectra is observed between the centre and outer regions of the coffee ring, indicating a more homogenous surface. When compared to the POPC only spectrum subtle differences can be observed in the POPC‐FML spectra in the wavenumber range 950–1800 cm^−1^ (Figure 7c). A broad alkyl CH_2_ peak at 1440 cm^−1^ can be observed in the complex spectra shifting from the sharper peak at 1463 cm^−1^ in the POPC only spectrum. As with the POPC‐ DFL complex the C−C stretch assigned peak at 1050 cm^−1^ has broadened and shifted to 1082 cm^−1^ indicating a change from *trans* to *gauche* confirmation with the addition of FML prior to drying. The appearance of a shoulder can also be observed at 1310 cm^−1^. The intensities of peaks in this region are sensitive to changes between *gauche* and *trans*.^[19]^ Similarly, in Figure 7d, a loss of peak intensity is observed for the CH_2_ asymmetric stretch at 2980 cm^−1^ and the CH_3_ methyl stretch with the appearance of CH_2_ symmetric stretch at 2852 cm^−1^ and 2926 cm^−1^ suggests that, as with DFP, the phospholipid chains are becoming more disordered with the addition of FML.

## Conclusion

In this work, Raman and NMR spectroscopy were used to report the physical properties of the ocular drugs DFP and FML in phospholipid bilayers. The Raman data suggest that both drugs decrease the order of the lipid chains, but the ^31^P NMR spectra of the lipid headgroups of hydrated bilayers indicate that the increased disorder does not disrupt the overall bilayer structure. The ability of the lipids to accommodate high levels of drug without disruption of the vesicular bilayer structure could be a favourable attribute for ocular therapy, where small volumes of liposomes may be required to deliver high drug concentrations over a small surface area.

The ^19^F NMR spectra of the drugs in the hydrated vesicles have lineshapes that confirm that the drugs reside fully in the lipid bilayer and are not distributed between the lipid and water phases, which may have implications for drug release kinetics. A novel feature of this work was the ability to determine the average orientation of DFP within the lipid bilayer, by exploiting the chemical shift and dipolar coupling parameters for the two fluorine nuclei in the molecule. The orientational preference of a molecule within cell membranes can influence its cellular uptake and may also affect its interaction with efflux transporters that export drugs from the cell.^[20]^

It should be noted that several assumptions are made in determining the drug orientation. The assumed order parameter *S*_mol_ of 0.8, which represents angular excursions of the rotational axis away from the bilayer normal, is taken from the value obtained for cholesterol in lipid bilayers. Deviations from this value by +/−0.1 units would affect the range of values of the calculated angle β only very slightly and so would not alter the determined orientation significantly. It is also assumed that the optimised structure of DFP represents its conformation in the membrane. The NMR spectra of the solid drug confirms that it can adopt multiple conformations and in the membrane environment the drug will sample different ring conformations in dynamic equilibrium. The conformational exchange is rapid on the NMR time scale and so the spectra reflect the time‐averaged conformation of the drug. Interestingly, FML adopts two principal orientations in the bilayers as compared with the single average orientation of DFP. The ability of FML to adopt two orientations may be attributed to the shorter aliphatic tail of the molecule, which is less effective at stabilising the molecule in the bilayer than are the longer tails of DFP.

The MLV samples for Raman analysis were dried to produce films and this process appears to result in lipid‐free POPC diffusing away from lipids associated with DFP. By contrast, FML remains associated with the lipid across the entire sample. The ability of Raman to detect these different physicochemical properties of the dried formulations could provide a useful tool for drug‐liposome quality control, as sample drying during formulation is employed to increase the stability of the drug product for storage.^[21]^ Air drying or lyophilisation of liposomes in the absence of cryoprotectants such as trehalose is known to have several effects upon the lipids.^[22]^ Although a lamellar‐like structure is preserved, the phase transition temperature increases due to increased van der Waals interactions between the lipids after removal of water from the headgroup region. This can cause leakage of the drug cargo from liposomes and upon rehydration the liposomes can coalesce to form larger structures.^[22]^ It was not possible to visualise the morphology of the dried vesicles on the steel slides used for Raman analysis, but analysis of pre‐dried and rehydrated MLVs by negative‐stain transmission electron microscopy indicated that the vesicular morphology is preserved after drying on carbon‐coated copper grids (Figure S5), with a size range (<2 μm) that is typical for MLVs. Dehydration damage to the MLVs on the steel slides for Raman cannot be ruled out, however. That FML, but not DFP, associates uniformly with the lipids is surprising because DFP is the more lipophilic drug, having a higher logP(octanol/water) (3.4 compared to 2.0). The extent of drug and lipid colocalisation may simply reflect the effectiveness with which the drugs stabilise the vesicles during the drying process. Further Raman experiments, beyond the scope of this work, could investigate whether cryoprotectants help to maintain a uniform distribution of lipids and drug.

In summary, the combined use of Raman and ^19^F solid‐state NMR spectroscopy has revealed insights into the orientation, distribution and lipid‐chain perturbing effects of a fluorinated ocular drug within phospholipid bilayers. The use of ^19^F NMR is attractive because there is no background signal, the spectra can be assigned unambiguously to the drug and ∼30 % of licensed drugs contain one or more fluorine atoms, making the methodology widely applicable. The wealth of information provided by the two techniques combined could be correlated with drug release kinetics to optimize the formulation of drug‐liposomal complexes for delivery to the eye and other tissue.

## Experimental Section

### Preparation of membrane samples

Solid DFP (Sigma Aldrich) was analysed as received without recrystallisation. For vesicle preparation, the compound (5 mg) was dissolved in chlorofom: methanol (50 : 50) with a 10‐fold molar excess of the lipid 1‐palmitoyl‐2‐oleoyl‐sn‐glycero‐3‐phosphocholine (POPC) and dried to a thin film in a round bottom flask under nitrogen and then under high vacuum. Multilamellar vesicles (MLVs) were produced by resuspending the film in water, subjecting to 5 freeze‐thaw cycles. For Raman spectroscopy 30 μL of the sample were pipetted onto a stainless‐steel slide and dried at room temperature. For NMR analysis the samples were centrifuged in a bench top centrifuge to remove excess liquid and the pellet was transferred to a 3.2 mm diameter zirconium rotor.

### NMR analysis

All measurements were performed on a Bruker Avance III spectrometer with an 89 mm bore magnet operating at 9.3 T, at a sample temperature of 30 °C. A Bruker quadruple resonance (HFXY) magic‐angle spinning probe tuned simultaneously to ^19^F and ^1^H was used for all measurements. The proton‐decoupled ^19^F CP‐MAS NMR spectrum of solid DFP was obtained with 5 kHz sample spinning. An initial 2.5 μs 90° pulse on ^1^H was followed by 2 ms ramped cross‐polarization from ^1^H to ^19^F at a proton nutation frequency of 40 kHz followed by irradiation of protons at a field of 83 kHz during signal acquisition. The spectrum is the result of averaging 512 transients with a recycle delay of 5 s. The proton‐decoupled ^19^F NMR spectra of the membrane samples were obtained with direct excitation of ^19^F using a 4.2 μs 90° pulse. The spectrum of the hydrated sample is the result of averaging 1600 transients with a recycle delay of 2 s. The proton‐decoupled ^31^P spectra of the membrane samples were obtained in a flat‐coil probe, with a 4 μs 90° pulse followed by signal acquisition with 20 kHz proton decoupling.

### Computational details

The principal values of the ^19^F chemical shift tensor were obtained by least‐squares fitting of a multi‐component simulated spectrum using the Bruker Topspin function Sola. Optimisation of the molecular geometry and calculation of the NMR parameters was performed using the CASTEP density functional theory code,^[23]^ employing the GIPAW algorithm,^[24]^ which allows the reconstruction of the all‐electron wave function in the presence of a magnetic field. The CASTEP density functional theory calculations employed the generalised gradient approximation (GGA) PBE functional^[25]^ and core–valence interactions were described by pseudopotentials.^[26]^ In the geometry optimisation, all atomic positions were allowed to vary and the Grimme G06 semi‐empirical dispersion correction scheme was used.^[26]^ Calculations were performed using a planewave energy cut‐off of 50 Ry (680 eV) and due to the large cell size, a single k‐point at the fractional coordinate (0.25, 0.25, 0.25) in reciprocal space for integration over the Brillouin zone. The calculations generate the absolute shielding tensor (**σ**) and diagonalisation of the symmetric part of **σ** yields as eigenvalues the principal components σ_11_, σ_22_ and σ_33_ and their orientations in the molecular frame are given by the eigenvectors.

The angles defining the orientations of the ^19^F chemical shift tensor and ^1^H‐^19^F dipolar vectors relative to a given axis of rotation were calculated using a C program written specifically for the purpose. Briefly, the angles α_MR_ and β_MR_ defining the orientation of the axis of rotation in a fixed molecular reference frame were transformed into angles α_FR_ and β_FR_ defining the orientation of the rotation axis relative to the F28 and F29 chemical shift principal axes, and angle θ defining the orientation relative to each dipolar vector. The dynamically averaged chemical shift anisotropies, Δδ_av_, for each orientation were calculated according to Eq. [1] and compared with the measured values. The difference between the experimental and calculated values were calculated as a combined sum‐of‐squares (SS) from the equation

(3) $$SS\left(\alpha_{MR},\beta_{MR}\right)={\left(C_{F28}-M_{F28}\right)}^{2}+{\left(C_{F29}-M_{F29}\right)}^{2}$$

where *C* and *M* are the calculated and measured values of Δδ_av_ for F28 and F29. Values of angles α_MR_ and β_MR_ were considered to be consistent with the data when SS<5.0 ppm. The proton‐coupled ^19^F NMR line shapes were simulated using SIMPSON.^[27]^ The exchange‐modulated lineshape simulations for FML were calculated for each orientation in the powder ensemble using the Bloch‐McConnell approach for each crystallite.

#### Raman spectroscopy

Raman spectra were acquired using a confocal Raman system (InVia, Renishaw plc, Wotton‐Under edge, UK) coupled to a 785 nm wavelength laser from 850–3000 cm^−1^. All spectra was acquired using 50× objective with a laser power at the sample of ∼5 mW, exposure time of 10 s. Spectra of DFP were collected from the solid sample and the spectra of the membrane samples were collected from thin films produced from drying 30 μL pipetted onto stainless steel slides. Eight repeat spectra were collected and averaged for all samples. Spectral data was divided into the two spectral regions of interest 2800–3000 cm^−1^ and 850–1800 cm^−1^ before data processing and analysis. All data processing was carried out using Matlab (version R2016a) using in house toolboxes. After cosmic spike removal data were normalised using standard normal variate (SNV) before smoothing using a triangular average.

## Conflict of interest

The authors declare no conflict of interest.

## Supporting information

As a service to our authors and readers, this journal provides supporting information supplied by the authors. Such materials are peer reviewed and may be re‐organized for online delivery, but are not copy‐edited or typeset. Technical support issues arising from supporting information (other than missing files) should be addressed to the authors.

Supporting Information
