# Supplementary material for: 19F Solid‐State NMR and Vibrational Raman Characterization of Corticosteroid Drug‐Lipid Membrane Interactions
Source: Chempluschem. 2021 Nov 2;86(11):1517–23. doi: 10.1002/cplu.202100385 (PMC9297973; doi:10.1002/cplu.202100385)
Supplement: Supplementary file 1 — Supporting Information [file CPLU-86-1517-s001.pdf]

ChemPlusChem

Supporting Information

^{19}F Solid-State NMR and Vibrational Raman Characterization of Corticosteroid Drug-Lipid Membrane Interactions

Bethany Mapley, David Townsend, John Griffin, Lorna Ashton, and David A. Middleton*

Additional Methods

***Transmission Electron Microscopy*.** 100 µL samples of POPC (500 µM) incubated in 50 mM Tris buffer pH 8 at 37 ˚C overnight with gentle agitation. Carbon coated copper grids (Agar Scientific) were glow-discharged before 3 µL of suspended fibrils were deposited onto the grid. After 2 minutes the excess sample was removed via blotting with filter paper. Followed by x3 wash steps with 35 µL dH_2O drops on parafilm in quick succession, with blotting in between each step. The grid was negatively stained by inverting onto 3 drops of 2 % (w/v) uranyl acetate (UA) on parafilm in quick succession with 2 minutes on the last drop of UA, and the excess stain blotted after each drop. The sample grid was dried under a heat lamp before imaging. The materials and microscope used were provided. The sample grid was imaged using a FEI Tecnai T12 microscope with a 120 keV Lab6 electron source and a Gatan US4000/SP 4k x 4k CCD camera, at 68 k magnification.

Additional results

Raman "coffee ring" comparison for pure POPC. The middle and edge of all dried samples were analysed to investigate the possibility of the formation of ring-like patterns, attributed to a phenomenon called the coffee-ring effect. This process can occur when a drop of any fluid containing non-volatile compounds is left on a flat surface to dry and results in a residue being left along the perimeter of the drops contour. For the pure POPC samples no difference in spectra were observed across the coffee ring. Example spectra from the centre and outer areas of the dried sample are shown in Figure S1

^{19}F CP-MAS spectra of solid DFP. The spectra of solid DFP at a spinning frequency of 5 kHz (Figure S3) reveal more peaks than expected for the two fluorine environments of the drug. For F29 of DFP there are 5 discernable peaks, spanning 8-9 ppm, whereas for F28 the frequency range is much narrower (1-2 ppm). The frequency of F29 therefore appears to be more sensitive to different ring conformations than is the F28 frequency. The ^{19}F chemical shift tensor values for both fluorine spins were obtained with least-squares fitting to the experimental spectrum, resulting in 2 sets of values for the F28 peaks and 5 sets of values for the F29 peaks (Tables S1 and S2). The average values were taken for the calculation of d_{iso}, d_{st} and η (Table S1).

Table S1. Accurate isotropic chemical shifts of F28 and F29 for the five detected forms of solid DFP resolved in the 2D ^{19}F-^{19}F dipolar correlation NMR spectrum.

Form	1	2	3	4	5
δ_{iso} (F28) (ppm)	-171.7	-171.6	-171.8	-172.2	-171.2
δ_{iso} (F29) (ppm)	-188.5	-189.8	-191.5	-192.2	-195.0

Table S2. Summary of the ^{19}F chemical shift values for solid difluprednate measured from the spectrum given in Figure S3 and calculated values obtained from DFT. Averages of the principal values taken from the multiple peaks (2 for F28 and 5 for F29) were used for the calculations of α and β according to Equation 1 of the main text.

	δ_{xx} (ppm)	δ_{yy} (ppm)	δ_{zz} (ppm)	δ_{iso} (ppm)	$\Delta\delta_{st}$ (ppm)	η
F28 (1)	-185.5	-176.5	-151.8			
F28 (2)	-186.8	-176.0	-152.6			
F28 (av.)	-186.2	-176.3	-152.2	-171.5	19.4	0.52
F28 (cal.)	-181.0	-170.0	-138.0	-163.0	25.0	0.44
F29 (1)	-168.0	-180.2	-217.2			
F29 (2)	-170.0	-181.4	-218.0			
F29 (3/4)	-170.1	-182.5	-222.5			
F29 (5)	-172.0	-185.0	-228.1			
F29 (av.)	-170.0	-182.3	-221.5	-191.3	-30.2	0.41
F29 (cal.)	-160.0	-190.0	-231.0	-193.7	-37.3	0.80

Table S3. Summary of the ^{19}F chemical shift values for DFP in POPC membranes, measured from the spectrum given in Figure 3c. The principal values, δ_{xx}, δ_{yy} and δ_{zz}, were obtained by least-squares fitting, and used to calculate the isotropic chemical shift [$\delta_{iso} = (\delta_{xx} + \delta_{yy} + \delta_{zz})/3$], static anisotropy [$\Delta\delta_{st} = (\delta_{zz} - \delta_{iso})$] and asymmetry parameter [$\eta = (\delta_{yy} - \delta_{zz})/\Delta\delta_{st}$]. The convention $|\delta_{zz} - \delta_{iso}| \geq |\delta_{xx} - \delta_{iso}| \geq |\delta_{yy} - \delta_{iso}|$ is followed.

	δ_{xx} (ppm)	δ_{yy} (ppm)	δ_{zz} (ppm)	δ_{iso} (ppm)	$\Delta\delta_{av}$ (ppm)	η
F28	-168.43	-168.43	-170.20	-169.0	-1.18	0.0
F29	-185.85	-193.15	-193.15	-190.72	4.87	0.0

Table S4. Variation in the values of angles α_{MR} and β_{MR} obtained from Eq. [1] of the main text, as a function of the order parameter S_{mol}.

Orientation group	S_{mol} (deg.)	α_{MR} (deg.)	β_{MR} (deg.)
[1]			
	0.9	90 ± 90	0 ± 5
	0.8	90 ± 90	0 ± 6
	0.7	90 ± 90	0 ± 7
	0.6	90 ± 90	0 ± 8
[2]			
	0.9	50 ± 5	87 ± 5
	0.8	49 ± 6	87 ± 5
	0.7	51 ± 7	86 ± 7
	0.6	53 ± 9	84 ± 7
[3]			
	0.9	91 ± 6	74 ± 4
	0.8	89 ± 7	74 ± 5
	0.7	86 ± 7	74 ± 5
	0.6	84 ± 9	74 ± 6

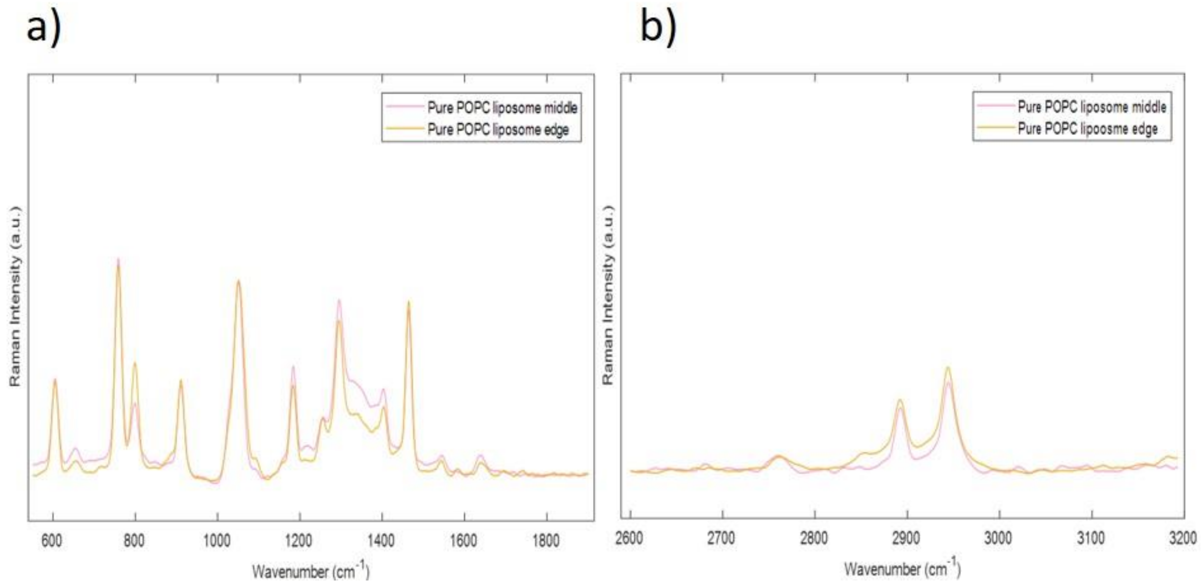

Figure S1. Averaged Raman spectra of the centre (n=3) and edge (n=3) regions of the pure POPC liposome dried sample at wavenumber ranges (a) 550-1900 cm^{-1} and (b) 2600-3200 cm^{-1}.

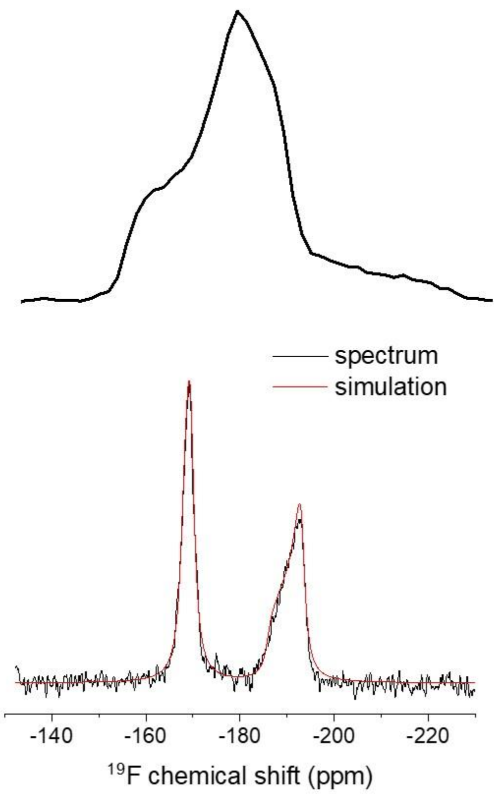

Figure S2. Comparison of the static ^{19}F NMR spectra of solid DFP (top) and DFP in POPC MLVs (bottom).

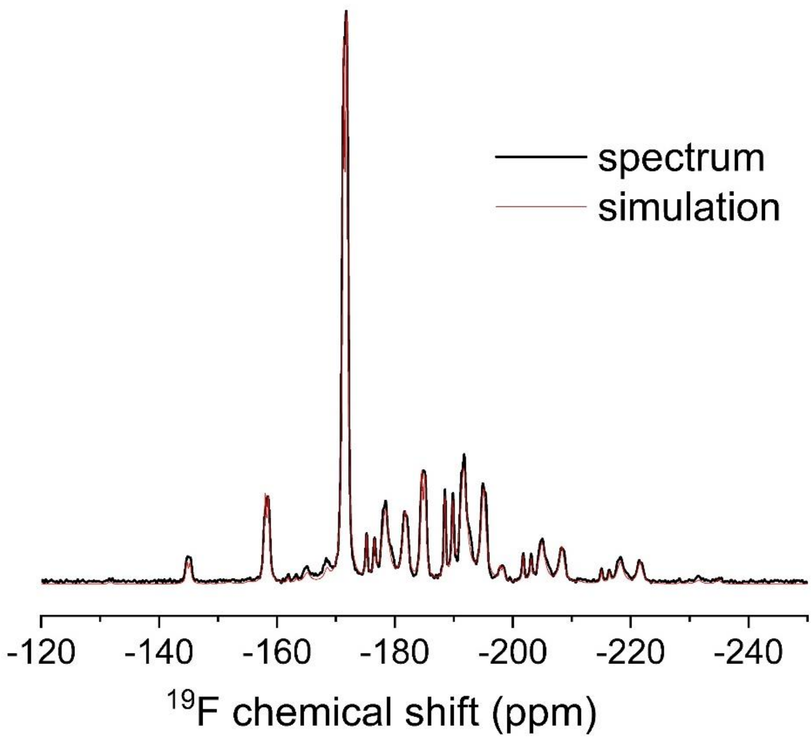

Figure S3. Proton-decoupled ^{19}F cross-polarization magic-angle spinning (CP-MAS) spectrum of solid difluprednate obtained at a 5 kHz MAS frequency (black) overlaid with the best-fitting simulated spectrum (red) from which were obtained the chemical shift values in Table S1.

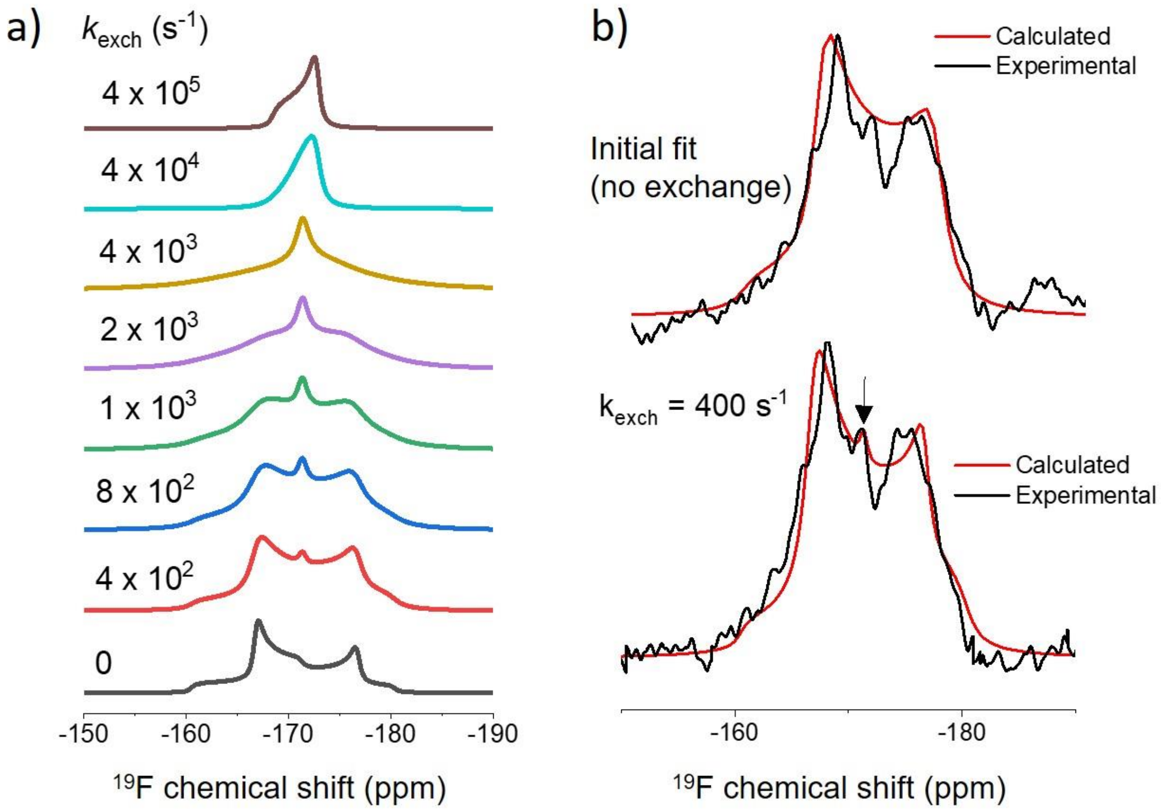

Figure S4. Analysis of the ^{19}F NMR spectrum of FML in POPC bilayers, including exchange between two orientations with rate constant k_{exch}. (a) Simulated spectra for different rate constants. The chemical shift parameters and α_{FR}, β_{FR} values for the two components of the lineshape are as given in the main text. (b) Comparison of the experimental spectrum with the initial simulated spectrum and after introducing an exchange rate constant of 400 s^{-1} into the simulation.

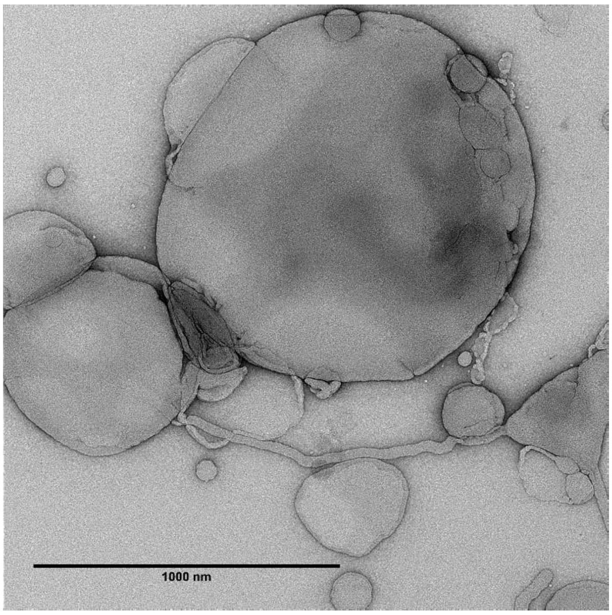

Figure S5. Uranyl acetate stained TEM image of POPC vesicles after drying and rehydration, before deposition on carbon-coated copper grids.
